# Epigenome Wide Associations of Smoking Behavior in the Health and Retirement Study

**DOI:** 10.1093/geroni/igab046.2509

**Published:** 2021-12-17

**Authors:** Jonah Fisher, Colter Mitchell, Helen Meier, Eileen Crimmins, Bharat Thyagarajan, Jessica Faul

**Affiliations:** 1 University of Michigan, Ann Arbor, Michigan, United States; 3 University of Southern California, Los Angeles, California, United States; 4 University of Minnesota, Minneapolis, Minnesota, United States

## Abstract

DNA methylation (DNAm) is an increasingly popular biomarker of health and aging outcomes. Smoking behaviors have a significant and well documented correlation with methylation signatures within the epigenome and are important confounding variables to account for in epigenome-wide association studies (EWAS). However, the common classification of individuals as ‘current’, ‘former’, and ‘never’ smokers may miss crucial DNAm patterns associated with other smoking behaviors such as duration, intensity, and frequency of cigarette smoking, resulting in an underestimation of the contribution of smoking behaviors to DNAm and potentially biasing EWAS results. We investigated associations between multiple smoking behavioral phenotypes (smoking pack years, smoking duration, smoking start age, and smoking end age) and single site DNAm using linear regressions adjusting for age, sex, race/ethnicity, education, and cell-type proportions in a subsample of individuals who participated in the HRS 2016 Venous Blood Study (N=1,775). DNAm was measured using the Infinium Methylation EPIC BeadChip. All 4 phenotypes had significant associations (FDR < 0.05) with many methylation sites (packyears=6859, smoking duration=6572, start age=11374, quit age=773). There was not much overlap in DNAm sites between the full set of models with only 6 overlapping between all 4. However, the phenotypes packyears and smoking duration showed large overlap (N=3782). Results suggest additional smoking phenotypes beyond current/former/never smoker classification should be included in EWAS analyses to appropriately account for the influence of smoking behaviors on DNAm.

